# Complete genome sequence of the *Vibrio vulnificus* strain VV2014DJH, a human-pathogenic bacterium isolated from a death case in China

**DOI:** 10.1186/s13099-017-0216-7

**Published:** 2017-11-21

**Authors:** Junhang Pan, Yi Sun, Wenwu Yao, Haiyan Mao, Yanjun Zhang, Muyuan Zhu

**Affiliations:** 1 College of Life Sciences, Zhejiang University, Hangzhou, 310058 China; 2 Zhejiang Provincial Center for Disease Control and Prevention, Hangzhou, 310051 China

## Abstract

**Background:**

*Vibrio vulnificus*, an opportunistic pathogen, is the causative agent of life-threatening septicemia and severe wound infections. However, the pathogenicity and virulence factors of *V. vulnificus* are not fully understood. Here we report the complete genome sequence of *V. vulnificus* VV2014DJH, which was isolated from a death case.

**Results:**

The genome of the *V. vulnificus* VV2014DJH contains two circular chromosomes with a mean G+C content of 46.8%, but does not consists of any plasmids. The chromosome I and chromosome II consist of 3,303,590 and 1,770,972 bp, respectively. In addition, the genome consists of 4617 protein coding genes, 172 RNA genes and type I, II and III secretion systems were predicted.

**Conclusions:**

In this study, the genomic information of the *V. vulnificus* VV2014DJH has been described. The information would contribute to the increasing scope and depth of *Vibrio* genome database, and provide insights into the pathogenicity and virulence factors of *V. vulnificus*.

**Electronic supplementary material:**

The online version of this article (10.1186/s13099-017-0216-7) contains supplementary material, which is available to authorized users.

## Introduction

*Vibrio vulnificus* is an opportunistic Gram-negative pathogen, which is widely distributed in marine environments around the world and has been isolated form water, sediments and seafood [1]. *V. vulnificus* is the leading cause of deaths reported from seafood in the United States with approximately 40 cases reported per year, and the fatality rate exceeds 50% [2]. However, the mortality rate ranges from 18 to 56% in China [3, 4]. In the recent years, more cases of amputation and death caused by *V. vulnificus* infections were reported in China, and most of these cases occurred in the coastal area of Zhejiang province [5]. In this paper, we report the complete genome sequence of *V. vulnificus* strain VV2014DJH, which was isolated from the blood culture of the death case of a shellfish aquaculture worker in Taizhou, Zhejiang province [6]. Determination of the genomic information of *V. vulnificus* strain VV2014DJH is necessary to understand the pathogenesis of the strain. Our study was approved by the ethics committee of the Zhejiang Provincial Center for Disease Control and Prevention (ZJCDC), China.

## Methods

The genomic DNA of VV2014DJH was extracted using the DNeasy Blood and Tissue Kit (QIAGEN, Germany) for Gram-negative bacteria, according to the manufacturer’s instructions. Total DNA was subjected to quality control by 2% agarose gel electrophoresis and quantified by a NanoDrop™ spectrophotometer. The sequencing of VV2014DJH was performed on Pacbio RS with Single Molecule, Real-Time (SMRT) technology. SMRT Analysis 2.3.0 was used to filter low quality reads, whose quality scores were under 40, and the filtered reads were assembled by SOAPdenovo2 to generate one contig without gaps [7]. According to Clusters of Orthologous Group (COG) category, circular representations with the annotated genes were obtained using Circos software and displayed the diagram of the VV2014DJH genome.

Transfer RNA (tRNA) genes were predicted by tRNAscan-SE [8]. Ribosome RNA (rRNA) genes were predicted with rRNAmmer [9] and sRNAs were obtained by BLAST against Rfam database [10]. RepeatMasker [11] (http://www.repeatmasker.org/) was used to detect repetitive sequences, and Tandem Repeat Finder [12] (http://tandem.bu.edu/trf/trf.html) was used to find Tandem Repeats. Gene prediction was performed on the VV2014DJH genome assembly by GeneMarkS [13] (http://topaz.gatech.edu/) with integrated model which combine the GeneMarkS generated (native) and Heuristic model parameters.

A whole genome BLAST [14] search (E-value less than 1E−5, minimal alignment length percentage larger than 40%) was performed against five databases. They are KEGG [15–17] (Kyoto Encyclopedia of Genes and Genomes), COG [18, 19], NR (Non-Redundant Protein Database), Swiss-Prot [20], and GO [21] (Gene Ontology) [22]. Secretory proteins were detected in the genome assembly by SignalP [23]. Type I–VII secretion system related proteins were extracted from all the annotation results. Type III secretion system effector proteins were detected by EffectiveT3 also [24]. The MUMmer system (version 3.0) was used applied for colinearity analysis of to compare the genome sequences of VV2014DJH, with CMCP6 and YJ016 [25]. The virulence factors annotation in VV2014DJH was performed by blasting the genome sequence to the Virulence factors Pathogenic Bacteria database (VFDB, http://www.mgc.ac.cn/cgi-bin/VFs/jsif/main.cgi) [26].

## Quality assurance

The genomic DNA used for sequencing was isolated from a single colony of the VV2014DJH. The 16S rRNA gene was sequenced and BLAST was performed against the NCBI database. In addition, the raw read sequences were selected and assembled only when their quality scores were more than 40 as cutoffs.

## Results

A PacBio RS sequencing run resulted in 56,579 reads with a mean read length of 8107 bp and a N50 read length of 11,532 bp. PacBio RS reads were assembled into one polished contigs with a N50 contig length of 3,307,273. The genome of VV2014DJH has a size of 5,074,562 bp with a mean G+C content of 46.8% and is composed of two circular chromosomes (chromosome I: 3,303,590 bp, 46.6% G+C content; chromosome II: 1,770,972 bp, 47.3% G+C content). It does not contain any plasmids.

**Fig. 1:**
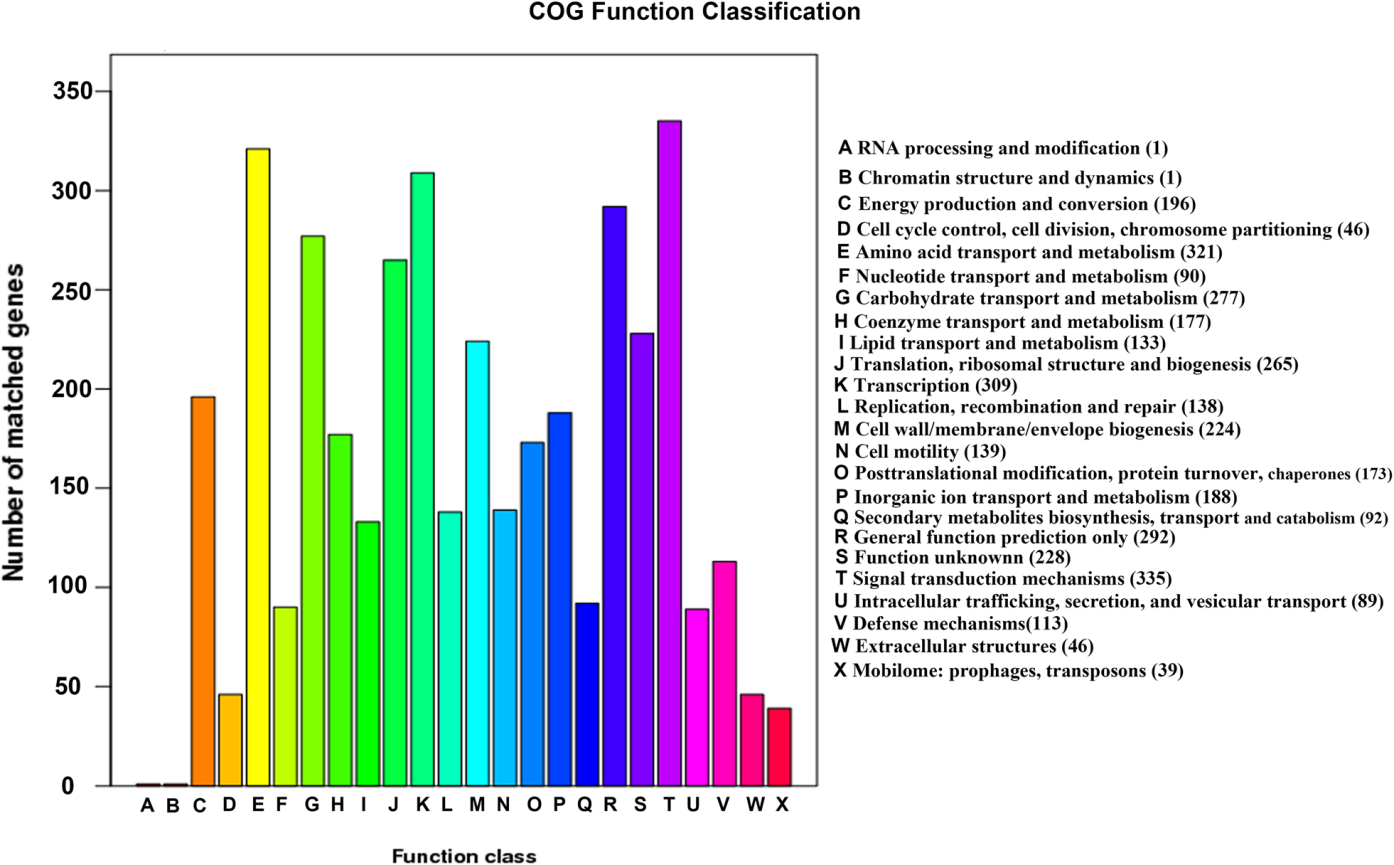
Functional classification of the protein coding genes (COG classification)

**Fig. 2:**
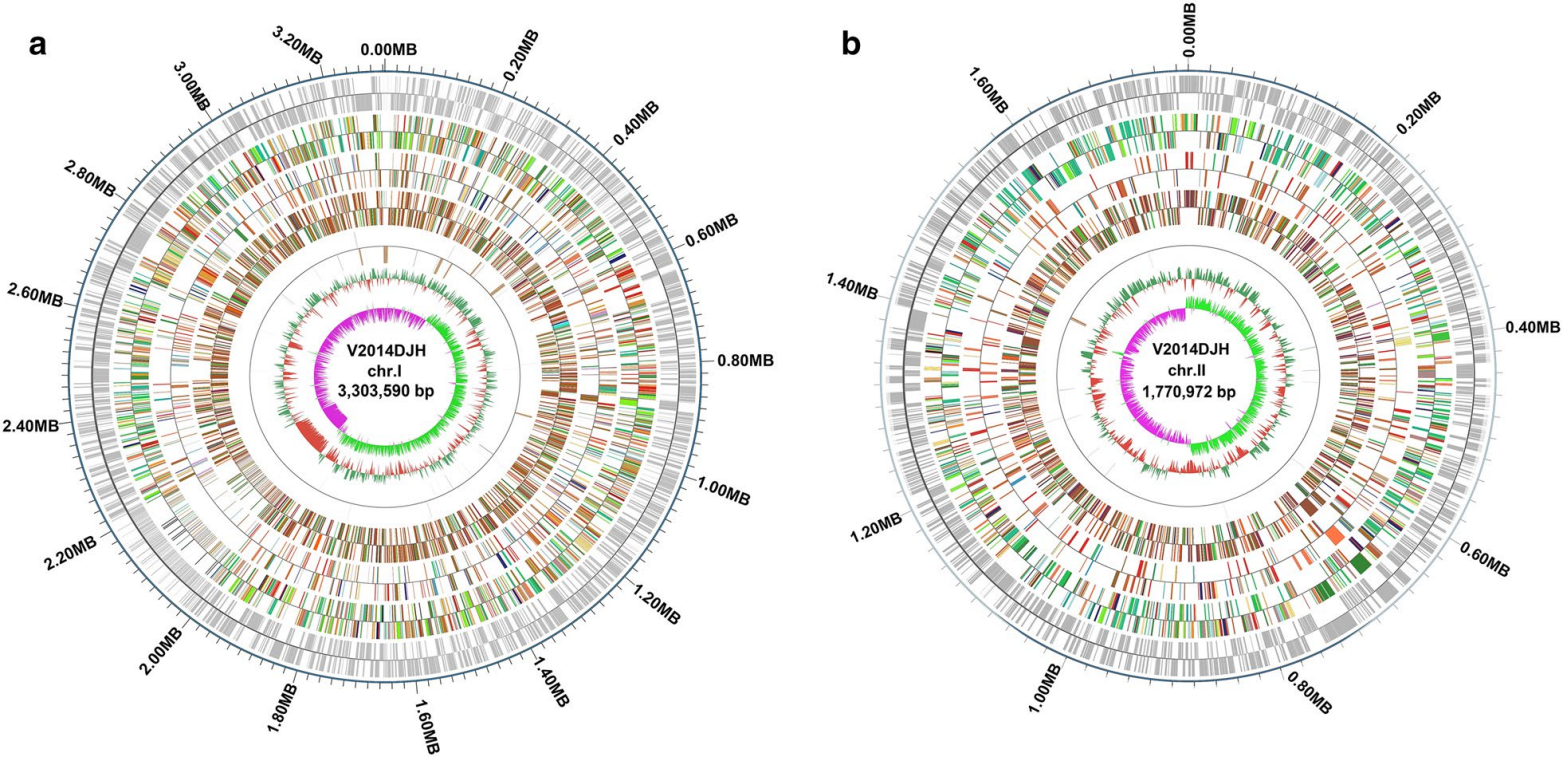
Circular representations of the *V. vulnificus* strain VV2014DJH chromosomes. **a** Chromosome I and **b** chromosome II. From the outside to the center: the coding genes, gene function annotation results (COG, KEGG, GO), ncRNA genes, genomic G+C content, genome GC skew value distribution

A total of 4617 protein coding genes and 172 RNA genes were predicted, and the general features of strain VV2014DJH are summarized in Tables 1, 2 and 3. The putative functions of the majority of the protein coding genes were prognosticated. The distribution of these genes into COGs functional categories was showed in Fig. 1, and the schematic circular representation of the VV2014DJH genome was created by Circos [27] in Fig. 2 to show the annotation information.

**Table 1:** General features of strain VV2014DJH genome

Type	Number
tRNA	120
5S rRNA	13
16 S rRNA	12
23 S rRNA	12
sRNA	15
Protein coding genes	4617

**Table 2:** Interspersed repetitive sequences

Type	Number	Total length (bp)	In genome (%)	Average length (bp)
LTR	121	10,795	0.213	89
DNA	38	2554	0.050	68
LINE	40	2442	0.048	64
SINE	26	2291	0.045	90
RC	3	164	0.003	55
Unknown	1	98	0.002	98

**Table 3:** Tandem repetitive sequences

Type	Number	Repeat size (bp)	Total length (bp)	In genome (%)
TR	157	6–801	41,505	0.818
Minisatellite	72	11–51	3113	0.061
Microsatellite	1	6	124	0.002

Three hundred and fifty-seven secretory proteins were detected in the 4617 proteins, and 45 proteins related to type I, II, III, IV and VI secretion systems were predicted, no protein related to type V and VII secretion systems was found. Type III secretion systems are complex bacterial structures that provide Gram-negative pathogens with unique virulence mechanisms. In the strain VV2014DJH, 184 effector proteins were detected by EffectiveT3 software.

The results of colinearity analysis for these three genome sequences of VV2014DJH, CMCP6 and YJ016 showed that the majority of the genomes were conserved in all three strains, although these were some transpositions between VV2014DJH and CMCP6, or VV2014DJH and YJ016. The Blast results showed that the pathogenic factors of VV2014DJH including the aforementioned type II and type VI secretion systems, flagellin C/D/E, and other pathogenic factors were also found in *V. vulnificus* VV2014DJH (Additional file 1: Fig S1, Table S1).

## Significance

Our report provides an extended understanding on *Vibrio vulnificus* at genomic level and may provide useful information for epidemiological investigation, pathogenicity and virulence factors. In addition, the genome information is probably helpful for rapid detection and prevention of food poisoning by the strain.

## Additional file

**Additional file 1: Figure S1.** Colinearity analysis of VV2014DJH, CMCP6 and YJ016. **Table S1.** The result of *Vibrio vulnificus* VV2014DJH blast against virulence factors of pathogenic bacteria database.
