# Supplementary material for: Complete genome sequence of the Vibrio vulnificus strain VV2014DJH, a human-pathogenic bacterium isolated from a death case in China
Source: Gut Pathog. 2017 Nov 21;9:67. doi: 10.1186/s13099-017-0216-7 (PMC5697068; doi:10.1186/s13099-017-0216-7)
Supplement: Supplementary file 1 — Additional file 1: Figure S1. Colinearity analysis of VV2014DJH, CMCP6 and YJ016. Table S1. The result of Vibrio vulnificus VV2014DJH blast against virulence factors of pathogenic bacteria database. [file 13099_2017_216_MOESM1_ESM.docx]

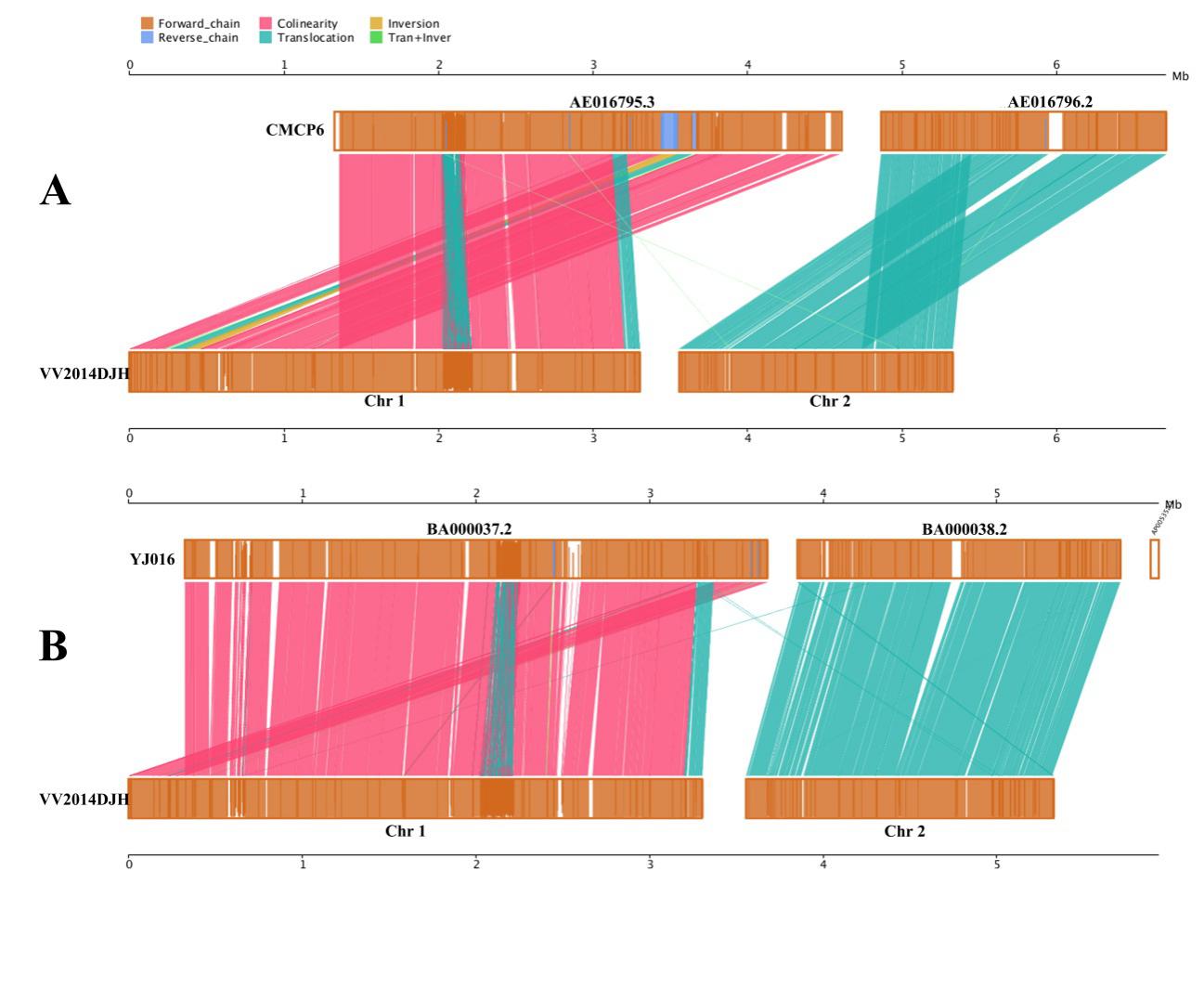


Figure S1. Colinearity analysis of VV2014DJH, CMCP6 and YJ016.

Table S1.The result of *Vibrio vulnificus* VV2014DJH Blast against Virulence Factors of Pathogenic Bacteria database

| Gene_id | Identity | E_value | VFDB_internal_id | VF_id | VF_name | Related_genes |
| --- | --- | --- | --- | --- | --- | --- |
| V2014DJHGM000066 | 58.33 | 4.00E-145 | VFG2361 | VF0392 | O-antigen | galE - UDP-glucose 4-epimerase |
| V2014DJHGM000146 | 74.52 | 0 | VFG1855 | VF0159 | Hsp60 | htpB - Hsp60, 60K heat shock protein HtpB |
| V2014DJHGM000154 | 48.73 | 2.00E-65 | VFG1867 | VF0169 | SodB | sodB - superoxide dismutase |
| V2014DJHGM000462 | 40.63 | 1.00E-94 | VFG1249 | VF0273 | Flagella | fleR - two-component response regulator |
| V2014DJHGM000477 | 54.17 | 0 | VFG2047 | VF0333 | T2SS | gspD - putative type II secretion protein |
| V2014DJHGM000478 | 65.38 | 0 | VFG2048 | VF0333 | T2SS | gspE - putative type II secretion protein |
| V2014DJHGM000479 | 51.24 | 3.00E-144 | VFG2049 | VF0333 | T2SS | gspF - putative type II secretion protein |
| V2014DJHGM000480 | 74.32 | 2.00E-82 | VFG2050 | VF0333 | T2SS | gspG - putative type II secretion protein |
| V2014DJHGM000482 | 40.37 | 7.00E-17 | VFG2052 | VF0333 | T2SS | gspI - putative type II secretion protein |
| V2014DJHGM000497 | 40.17 | 1.00E-49 | VFG1390 | VF0298 | MprAB | mprA - hypothetical protein Rv0981 |
| V2014DJHGM000552 | 43.66 | 7.00E-40 | VFG0320 | VF0056 | LPS | kdtB - lipopolysaccharide core biosynthesis protein (kdtB) |
| V2014DJHGM000563 | 46.35 | 3.00E-102 | VFG1338 | VF0274 | Capsule | neuB - N-acetyl neuramic acid synthetase NeuB |
| V2014DJHGM000579 | 45.28 | 3.00E-117 | VFG0141 | VF0085 | LPS | waaA - lipopolysaccharide core biosynthesis protein WaaP |
| V2014DJHGM000580 | 52.19 | 2.00E-125 | VFG0143 | VF0085 | LPS | waaF - heptosyltransferase I |
| V2014DJHGM000582 | 75.64 | 3.00E-177 | VFG0332 | VF0044 | LOS | rfaD - ADP-L-glycero-D-mannoheptose-6-epimerase |
| V2014DJHGM000596 | 53.39 | 5.00E-137 | VFG1373 | VF0144 | Capsule | cps4I - UDP-N-acetylglucosamine-2-epimerase |
| V2014DJHGM000597 | 43.94 | 3.00E-116 | VFG1311 | VF0003 | Capsule | cap8O - capsular polysaccharide synthesis enzyme Cap8O |
| V2014DJHGM000606 | 51.05 | 2.00E-43 | VFG1309 | VF0003 | Capsule | cap8M - capsular polysaccharide synthesis enzyme Cap8M |
| V2014DJHGM000607 | 47.48 | 7.00E-179 | VFG0029 | VF0033 | LPS | bplL - lipopolysaccharide biosynthesis protein |
| V2014DJHGM000608 | 48.72 | 2.00E-143 | VFG1969 | VF0323 | Capsule | kfiD - putative UDP-glucose 6-dehydrogenase |
| V2014DJHGM000613 | 70.39 | 0 | VFG0106 | VF0127 | ACF | acfD - accessory colonization factor AcfD |
| V2014DJHGM000643 | 41.58 | 8.00E-51 | VFG1864 | VF0153 | Mip | mip - macrophage infectivity potentiator (Mip) |
| V2014DJHGM000686 | 48.06 | 1.00E-93 | VFG1971 | VF0323 | Capsule | kpsF - KpsF protein |
| V2014DJHGM000770 | 66.52 | 0 | VFG0331 | VF0044 | LOS | rfaE - ADP-heptose synthase |
| V2014DJHGM000790 | 40.43 | 4.00E-47 | VFG2026 | VF0326 | LOS | gmhA - phosphoheptose isomerase |
| V2014DJHGM000897 | 46.71 | 0 | VFG0079 | VF0072 | ClpC | clpC - endopeptidase Clp ATP-binding chain C |
| V2014DJHGM000924 | 61.83 | 0 | VFG1381 | VF0253 | Isocitrate lyase | icl/aceA - aceA |
| V2014DJHGM001116 | 40.44 | 1.00E-30 | VFG2351 | VF0394 | Flagella | flgB - flagellar basal-body rod protein FlgB |
| V2014DJHGM001117 | 51.37 | 6.00E-48 | VFG1236 | VF0273 | Flagella | flgC - flagellar basal-body rod protein FlgC |
| V2014DJHGM001118 | 40.49 | 1.00E-42 | VFG1237 | VF0273 | Flagella | flgD - flagellar basal-body rod modification protein FlgD |
| V2014DJHGM001120 | 47.43 | 1.00E-74 | VFG1239 | VF0273 | Flagella | flgF - flagellar basal-body rod protein FlgF |
| V2014DJHGM001121 | 59.92 | 5.00E-109 | VFG1240 | VF0273 | Flagella | flgG - flagellar basal-body rod protein FlgG |
| V2014DJHGM001122 | 45.58 | 2.00E-56 | VFG1241 | VF0273 | Flagella | flgH - flagellar L-ring protein precursor FlgH |
| V2014DJHGM001123 | 62.72 | 4.00E-149 | VFG1242 | VF0273 | Flagella | flgI - flagellar P-ring protein precursor FlgI |
| V2014DJHGM001124 | 47.27 | 7.00E-44 | VFG1243 | VF0273 | Flagella | flgJ - flagellar protein FlgJ |
| V2014DJHGM001130 | 40.38 | 2.00E-25 | VFG2321 | VF0394 | Flagella | fliC2 - flagellin |
| V2014DJHGM001170 | 78.62 | 2.00E-85 | VFG0478 | VF0113 | Fur | fur - transcriptional repressor of iron-responsive genes (Fur family) (ferric uptake regulator) |
| V2014DJHGM001197 | 42.86 | 0 | VFG1859 | VF0160 | FeoAB | feoB - ferrous iron transporter B |
| V2014DJHGM001255 | 45.92 | 2.00E-61 | VFG2085 | VF0335 | VAS cluster | vasH - sigma-54 dependent transcriptional regulator |
| V2014DJHGM001258 | 62.18 | 2.00E-96 | VFG0077 | VF0074 | ClpP | clpP - ATP-dependent Clp protease proteolytic subunit |
| V2014DJHGM001311 | 41.94 | 2.00E-25 | VFG1386 | VF0286 | PhoP | phoP - phoP |
| V2014DJHGM001329 | 67.71 | 7.00E-100 | VFG1867 | VF0169 | SodB | sodB - superoxide dismutase |
| V2014DJHGM001347 | 40.97 | 7.00E-95 | VFG1214 | VF0082 | Type IV pili | pilR - two-component response regulator PilR |
| V2014DJHGM001384 | 48.91 | 8.00E-68 | VFG1889 | VF0262 | LetA/S | letA - response regulator GacA |
| V2014DJHGM001429 | 40 | 4.00E-10 | VFG0542 | VF0116 | TTSS(SPI-1 encode) | iacP - putative acyl carrier protein |
| V2014DJHGM001547 | 50.44 | 1.00E-67 | VFG0116 | VF0091 | Alginate | algB - two-component response regulator AlgB |
| V2014DJHGM001563 | 41.07 | 1.00E-73 | VFG1248 | VF0273 | Flagella | fleQ - transcriptional regulator FleQ |
| V2014DJHGM001565 | 40.64 | 1.00E-41 | VFG1206 | VF0272 | FbpABC | fbpC - iron(III) ABC transporter, ATP-binding protein |
| V2014DJHGM001577 | 40.18 | 2.00E-54 | VFG1390 | VF0298 | MprAB | mprA - hypothetical protein Rv0981 |
| V2014DJHGM001635 | 40.96 | 4.00E-61 | VFG0922 | VF0227 | Chu | chuU - Putative permease of iron compound ABC transport system |
| V2014DJHGM001661 | 76.07 | 0 | VFG1862 | VF0168 | KatAB | katB - catalase-peroxidase KatB |
| V2014DJHGM001664 | 40.21 | 2.00E-57 | VFG1206 | VF0272 | FbpABC | fbpC - iron(III) ABC transporter, ATP-binding protein |
| V2014DJHGM001738 | 40.91 | 7.00E-32 | VFG1369 | VF0144 | Capsule | cps4E - capsular polysaccharide biosynthesis protein Cps4E |
| V2014DJHGM001749 | 40.3 | 2.00E-97 | VFG1214 | VF0082 | Type IV pili | pilR - two-component response regulator PilR |
| V2014DJHGM001804 | 43.27 | 8.00E-66 | VFG1206 | VF0272 | FbpABC | fbpC - iron(III) ABC transporter, ATP-binding protein |
| V2014DJHGM001830 | 51.43 | 7.00E-69 | VFG1248 | VF0273 | Flagella | fleQ - transcriptional regulator FleQ |
| V2014DJHGM002404 | 42.86 | 8.00E-172 | VFG0080 | VF0073 | ClpE | clpE - ATP-dependent protease |
| V2014DJHGM002554 | 51.88 | 5.00E-84 | VFG1856 | VF0292 | CcmC | ccmC - heme exporter protein CcmC |
| V2014DJHGM002565 | 54.7 | 2.00E-87 | VFG2010 | VF0157 | Flagella | fliA - flagellar biosynthesis sigma factor FliA |
| V2014DJHGM002566 | 61.51 | 2.00E-112 | VFG1265 | VF0273 | Flagella | fleN - flagellar synthesis regulator FleN |
| V2014DJHGM002567 | 42.71 | 5.00E-73 | VFG2011 | VF0157 | Flagella | flhF - flagellar GTP-binding protein FlhF |
| V2014DJHGM002568 | 65.6 | 0 | VFG1263 | VF0273 | Flagella | flhA - flagellar biosynthesis protein FlhA |
| V2014DJHGM002569 | 50 | 4.00E-124 | VFG1262 | VF0273 | Flagella | flhB - flagellar biosynthetic protein FlhB |
| V2014DJHGM002570 | 44.75 | 4.00E-60 | VFG1261 | VF0273 | Flagella | fliR - flagellar biosynthetic protein FliR |
| V2014DJHGM002571 | 60 | 1.00E-32 | VFG1260 | VF0273 | Flagella | fliQ - flagellar biosynthetic protein FliQ |
| V2014DJHGM002572 | 66.67 | 2.00E-105 | VFG1259 | VF0273 | Flagella | fliP - flagellar biosynthetic protein FliP |
| V2014DJHGM002574 | 63.33 | 5.00E-51 | VFG1257 | VF0273 | Flagella | fliN - flagellar motor switch protein FliN |
| V2014DJHGM002575 | 60.75 | 3.00E-141 | VFG1256 | VF0273 | Flagella | fliM - flagellar motor switch protein FliM |
| V2014DJHGM002579 | 59.09 | 0 | VFG1254 | VF0273 | Flagella | fliI - flagellum-specific ATP synthase FliI |
| V2014DJHGM002581 | 58.15 | 2.00E-137 | VFG1252 | VF0273 | Flagella | fliG - flagellar motor switch protein FliG |
| V2014DJHGM002583 | 57.53 | 2.00E-27 | VFG2007 | VF0157 | Flagella | fliE - flagellar hook-basal body protein FliE |
| V2014DJHGM002584 | 53.28 | 2.00E-163 | VFG1249 | VF0273 | Flagella | fleR - two-component response regulator |
| V2014DJHGM002586 | 51.91 | 7.00E-154 | VFG1248 | VF0273 | Flagella | fleQ - transcriptional regulator FleQ |
| V2014DJHGM002587 | 40.48 | 3.00E-27 | VFG2325 | VF0394 | Flagella | fliS - flagellar protein FliS |
| V2014DJHGM002591 | 46.67 | 3.00E-37 | VFG2321 | VF0394 | Flagella | fliC2 - flagellin |
| V2014DJHGM002593 | 48.95 | 1.00E-64 | VFG1246 | VF0273 | Flagella | fliC - flagellin type B |
| V2014DJHGM002620 | 49.69 | 7.00E-50 | VFG2026 | VF0326 | LOS | gmhA - phosphoheptose isomerase |
| V2014DJHGM002707 | 45.99 | 2.00E-79 | VFG0088 | VF0126 | TCP | tcpI - toxin co-regulated pilus biosynthesis protein I |
| V2014DJHGM002740 | 58.54 | 7.00E-148 | VFG2361 | VF0392 | O-antigen | galE - UDP-glucose 4-epimerase |
| V2014DJHGM002843 | 40.62 | 1.00E-68 | VFG1206 | VF0272 | FbpABC | fbpC - iron(III) ABC transporter, ATP-binding protein |
| V2014DJHGM002862 | 56.15 | 7.00E-66 | VFG1417 | VF0319 | PanC/PanD | panC - panC |
| V2014DJHGM002876 | 42.86 | 9.00E-16 | VFG0221 | VF0075 | Type IV pili | pilE - pilin PilE |
| V2014DJHGM002877 | 53.02 | 0 | VFG1880 | VF0155 | Type IV pili | pilB - (type IV) pilus assembly protein PilB |
| V2014DJHGM002878 | 42.36 | 5.00E-113 | VFG0113 | VF0082 | Type IV pili | pilC - still frameshift type 4 fimbrial biogenesis protein PilC |
| V2014DJHGM002879 | 48.48 | 9.00E-47 | VFG1882 | VF0155 | Type IV pili | pilD - type 4 (IV) prepilin-like protein leader peptide processing enzyme PilD |
| V2014DJHGM002899 | 61.4 | 4.00E-23 | VFG1886 | VF0261 | CsrA | csrA - carbon storage regulator RsmA |
| V2014DJHGM002906 | 74.68 | 4.00E-170 | VFG0477 | VF0112 | RpoS | rpoS - sigma S (sigma 38) factor of RNA polymerase, major sigmafactor during stationary phase |
| V2014DJHGM002917 | 42.17 | 0 | VFG1887 | VF0260 | RelA | relA - GTP pyrophosphokinase ((p)ppGpp synthetase I) stringent stress response RelA |
| V2014DJHGM002933 | 63.87 | 1.00E-91 | VFG0121 | VF0091 | Alginate | algU - alginate biosynthesis protein AlgZ/FimS |
| V2014DJHGM002965 | 60.57 | 2.00E-157 | VFG1224 | VF0082 | Type IV pili | pilU - twitching motility protein PilU |
| V2014DJHGM002966 | 68.53 | 2.00E-180 | VFG1223 | VF0082 | Type IV pili | pilT - twitching motility protein PilT |
| V2014DJHGM003041 | 43.44 | 4.00E-106 | VFG2048 | VF0333 | T2SS | gspE - putative type II secretion protein |
| V2014DJHGM003051 | 41.95 | 1.00E-78 | VFG0964 | VF0244 | Hyaluronic acid capsule | hasC - UDP-glucose pyrophosphorylase |
| V2014DJHGM003096 | 70.44 | 0 | VFG1855 | VF0159 | Hsp60 | htpB - Hsp60, 60K heat shock protein HtpB |
| V2014DJHGM003131 | 56.97 | 1.00E-143 | VFG2361 | VF0392 | O-antigen | galE - UDP-glucose 4-epimerase |
| V2014DJHGM003214 | 43.74 | 0 | VFG0619 | VF0123 | Aerobactin | iutA - receptor-like protein iutA [Shigella flexneri (serotype 2a) 301] |
| V2014DJHGM003219 | 44.64 | 1.00E-68 | VFG0925 | VF0228 | Enterobactin | fepC - Ferric enterobactin transport ATP-binding protein fepC |
| V2014DJHGM003241 | 44.74 | 4.00E-63 | VFG1390 | VF0298 | MprAB | mprA - hypothetical protein Rv0981 |
| V2014DJHGM003258 | 50.5 | 2.00E-170 | VFG0146 | VF0087 | LasB | lasB - elastase LasB |
| V2014DJHGM003416 | 46.21 | 1.00E-153 | VFG1817 | VF0299 | Mycobactin | mbtA - mbtA |
| V2014DJHGM003417 | 55.45 | 2.00E-38 | VFG0170 | VF0095 | Pyochelin | pchB - salicylate biosynthesis protein PchB |
| V2014DJHGM003418 | 40.91 | 1.00E-77 | VFG0933 | VF0228 | Enterobactin | entB - Isochorismatase |
| V2014DJHGM003420 | 48.87 | 1.00E-179 | VFG0168 | VF0095 | Pyochelin | pchD - pyochelin biosynthesis protein PchD |
| V2014DJHGM003422 | 41.96 | 4.00E-64 | VFG0934 | VF0228 | Enterobactin | entA - 2,3-dihydro-2,3-dihydroxybenzoate dehydrogenase |
| V2014DJHGM003632 | 45.77 | 3.00E-36 | VFG1864 | VF0153 | Mip | mip - macrophage infectivity potentiator (Mip) |
| V2014DJHGM003645 | 45.66 | 5.00E-123 | VFG1089 | VF0264 | Hpt | hpt - highly similar to hexose phosphate transport protein |
| V2014DJHGM003673 | 41.77 | 0 | VFG0907 | VF0225 | Hemolysin | hlyB - Hemolysin B |
| V2014DJHGM003675 | 44.33 | 0 | VFG1269 | VF0028 | Cya | cyaB - cyclolysin secretion ATP-binding protein |
| V2014DJHGM003714 | 42.92 | 4.00E-56 | VFG2061 | VF0334 | HSI-I | PA0075 - probable phosphoprotein phosphatase |
| V2014DJHGM003716 | 40.99 | 6.00E-39 | VFG2069 | VF0334 | HSI-I | PA0083 - hypothetical protein |
| V2014DJHGM003717 | 51.8 | 1.00E-172 | VFG2070 | VF0334 | HSI-I | PA0084 - hypothetical protein |
| V2014DJHGM003723 | 45.42 | 0 | VFG2076 | VF0334 | HSI-I | clpV1 - probable ClpA/B-type chaperone |
| V2014DJHGM003853 | 40.76 | 2.00E-37 | VFG0869 | VF0215 | Dispersin | aatC - AatC ATB binding protein of ABC transporter |
| V2014DJHGM004005 | 49.45 | 2.00E-19 | VFG1306 | VF0003 | Capsule | cap8J - capsular polysaccharide synthesis enzyme Cap8J |
| V2014DJHGM004132 | 45 | 6.00E-64 | VFG0344 | VF0268 | HitABC | hitC - iron(III) ABC transporter, ATP-binding protein |
| V2014DJHGM004207 | 45.96 | 2.00E-69 | VFG0922 | VF0227 | Chu | chuU - Putative permease of iron compound ABC transport system |
| V2014DJHGM004332 | 49.71 | 2.00E-53 | VFG0463 | VF0109 | SodCI | sodCI - Gifsy-2 prophage: superoxide dismutase precursor (Cu-Zn) |
| V2014DJHGM004446 | 40.48 | 6.00E-75 | VFG1214 | VF0082 | Type IV pili | pilR - two-component response regulator PilR |
